# Infantile Parotid Hemangioma: A Challenging Diagnosis

**DOI:** 10.7759/cureus.86105

**Published:** 2025-06-15

**Authors:** Femke Vrijdag, Frédéric Claerhoudt, Marie-Sofie Walgraeve

**Affiliations:** 1 Radiology, AZ Sint-Jan Brugge, KU Leuven, Leuven, BEL; 2 Radiology, AZ Sint-Jan Brugge, UZ Gent, Ghent, BEL; 3 Radiology, AZ Sint-Jan Brugge, Brugge, BEL

**Keywords:** head and neck tumors, infantile parotid hemangioma, mri neck, neck ultrasound, parotid gland tumor

## Abstract

Infantile parotid hemangiomas (IPH) are the most prevalent infantile parotid tumors. Imaging features are key for diagnosis. The following report details the case of a female neonate who developed a rapidly enlarging left submandibular mass. Imaging findings on ultrasound and MRI revealed marked vascularity and characteristic flow voids, consistent with the diagnosis of IPH. The presence of two smaller cutaneous hemangiomas further supported the diagnosis. Treatment with propranolol resulted in complete regression of the parotid mass without complications. This case highlights the valuable role of imaging in the evaluation of paediatric neck masses. The ability to recognize the distinct imaging features of IPH on ultrasound and MRI allows for an accurate diagnosis and avoids unnecessary interventions. In addition, it is important to note that propranolol remains a safe and effective first-line treatment.

## Introduction

Infantile parotid hemangiomas (IPH), while rare, represent the most common infantile parotid tumor [1-5]. Female gender, low birth weight, and Caucasian race are risk factors [1,4,6]. Typical onset is before the age of four months [1-4]. Sometimes, a cutaneous component is present, making diagnosis obvious [1,3,6]. The lesion usually appears within a few weeks after birth and is mostly asymptomatic [1,3-5]. The clinical course is characterized by a proliferative phase, followed by a phase of involution, often spontaneously or under treatment, leading to complete resolution [1-5].

## Case presentation

**Figure 1:**
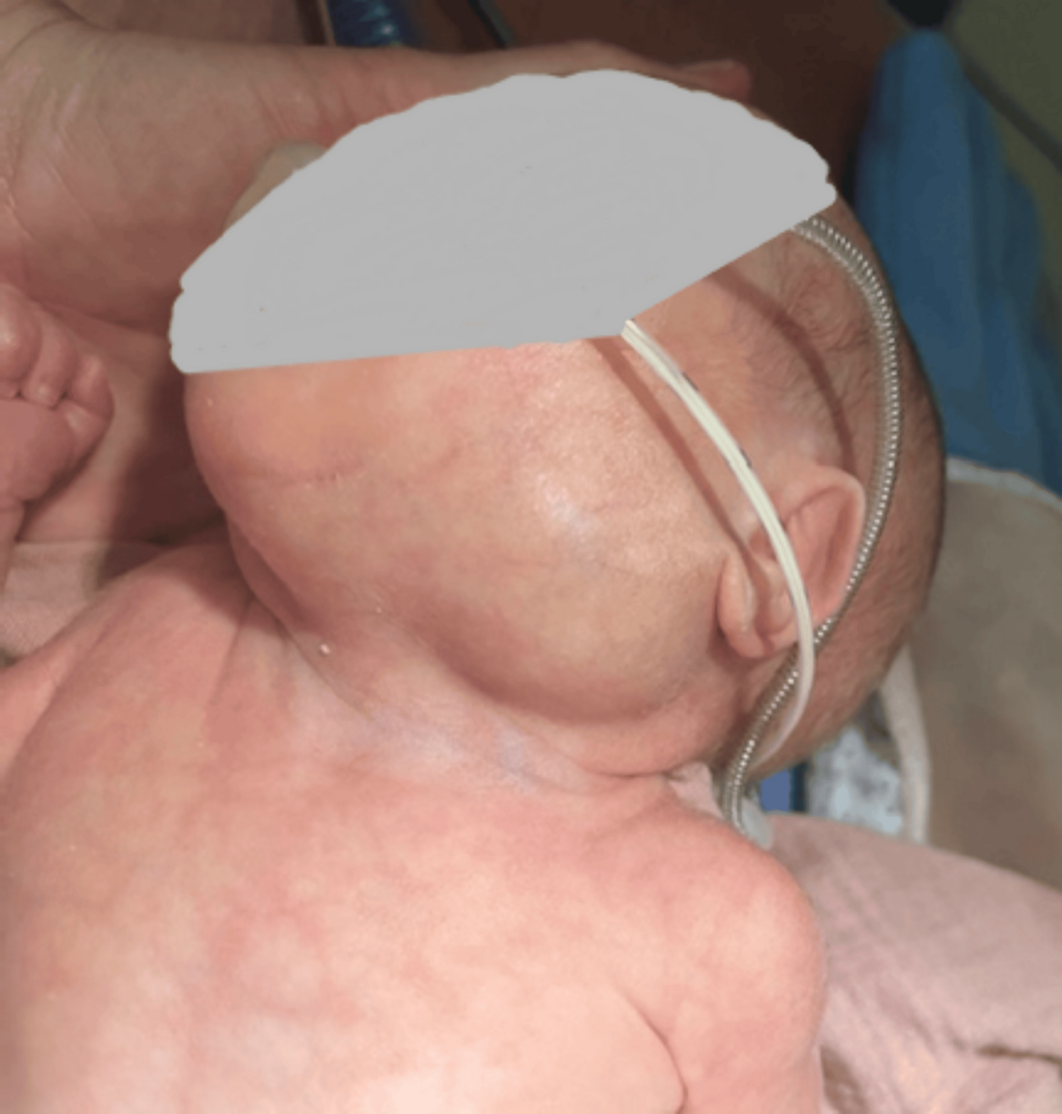
Evident mass in the left (sub)mandibular region, without abnormalities of the overlying skin. The mass was not present at birth, and suddenly appeared over the course of a few days, slowly increasing in size.

**Figure 2:**
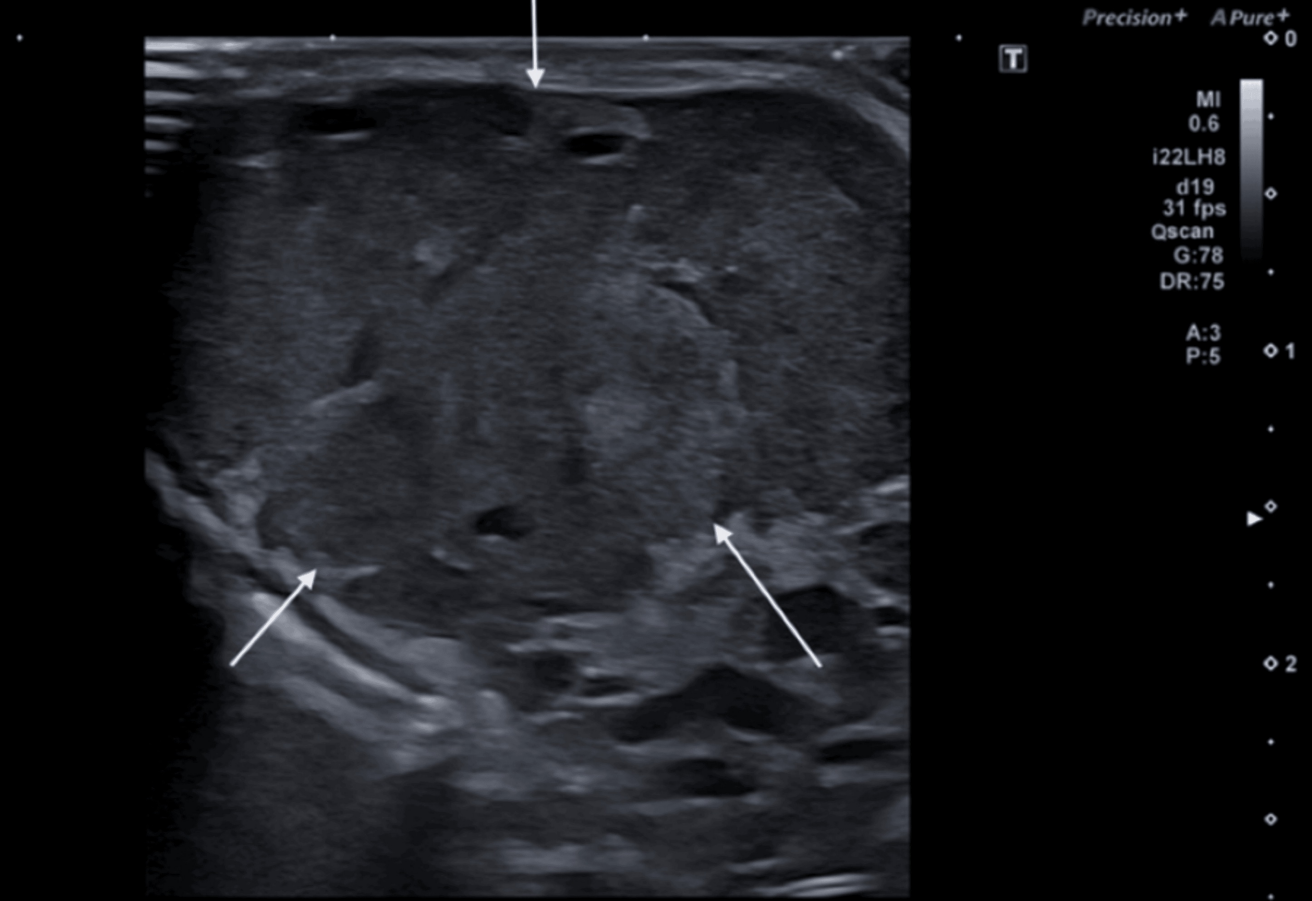
Ultrasound shows a sharply defined hypoechoic mass (white arrows) in the submandibular region.

**Figure 3:**
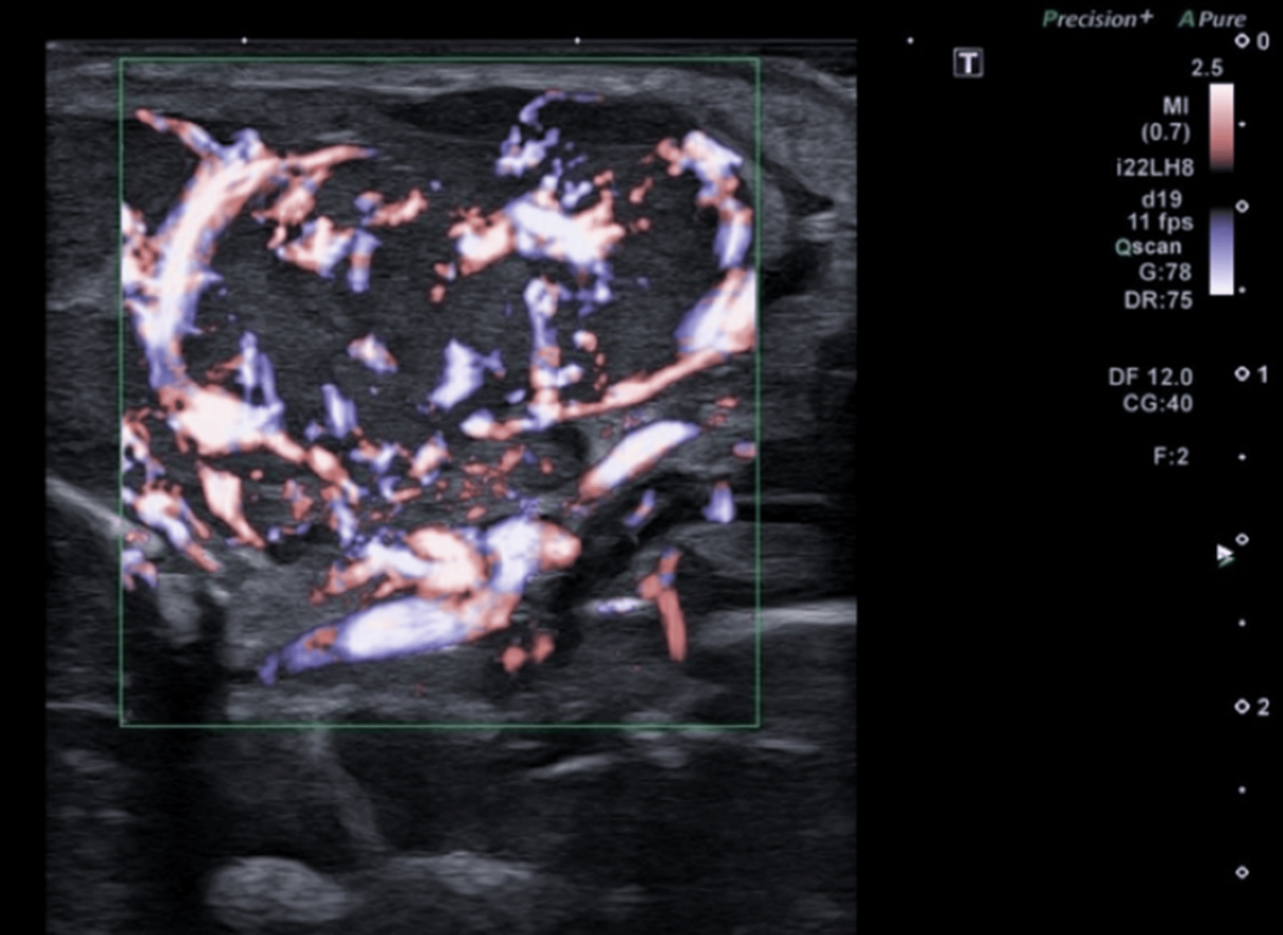
Doppler imaging of the lesion shows intense, diffuse vascularization.

A female neonate, born at GA of 28w4d, presented on the NICU six weeks after birth with a sudden swelling in the left submandibular/pre-auricular region. Physical examination revealed a soft, well-demarcated, round mass in the left submandibular region, covered with normal skin (Figure 1). Ultrasound demonstrated a sharply defined mass measuring 17 mm (Figure 2). Colour Doppler showed diffuse and intense flow within the mass, without evidence of necrosis or abscess (Figure 3). A tentative diagnosis of lymphadenitis (colli) was made, and the patient was initiated on antibiotics. However, blood samples revealed low inflammatory markers and viral serology for HIV, cytomegalovirus (CMV), Epstein-Barr virus (EBV), and Toxoplasmosis did not indicate acute infection. Nasopharyngeal aspirate results were negative for Mycoplasma, and hemocultures also yielded negative results. Because of the discrepancy between clinical and imaging findings, an MRI of the neck was performed for further evaluation. The treatment was discontinued approximately 10 months after the initial diagnosis. Subsequent follow-up, both clinical and ultrasound-based, is anticipated. If rebound growth occurs, the treatment will be reinitiated.

**Figure 4:**
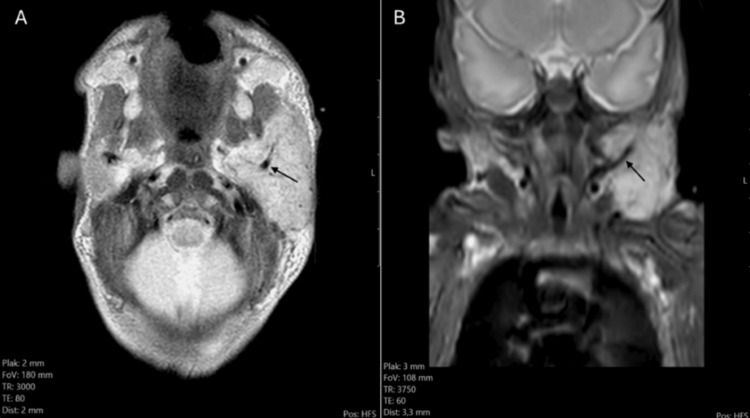
(A) axial T2 TSE image shows an enlarged left parotid, slightly hyperintense compared to the normal right parotid. Flow voids are visible (black arrow). (B) Coronal T2 Fatsat image shows the mass in the left mandibular region with internal flow voids (black arrow).

T2 TSE and T2 Fatsat images confirmed a mass in the left submandibular/pre-auricular region, comprising an enlarged left parotid gland measuring 40 mm x 22 mm x 33 mm (Figures 4A-4B). The mass was slightly T2 hyperintense compared to the normal parotid gland on the right side, with visible intralesional flow voids.

**Figure 5:**
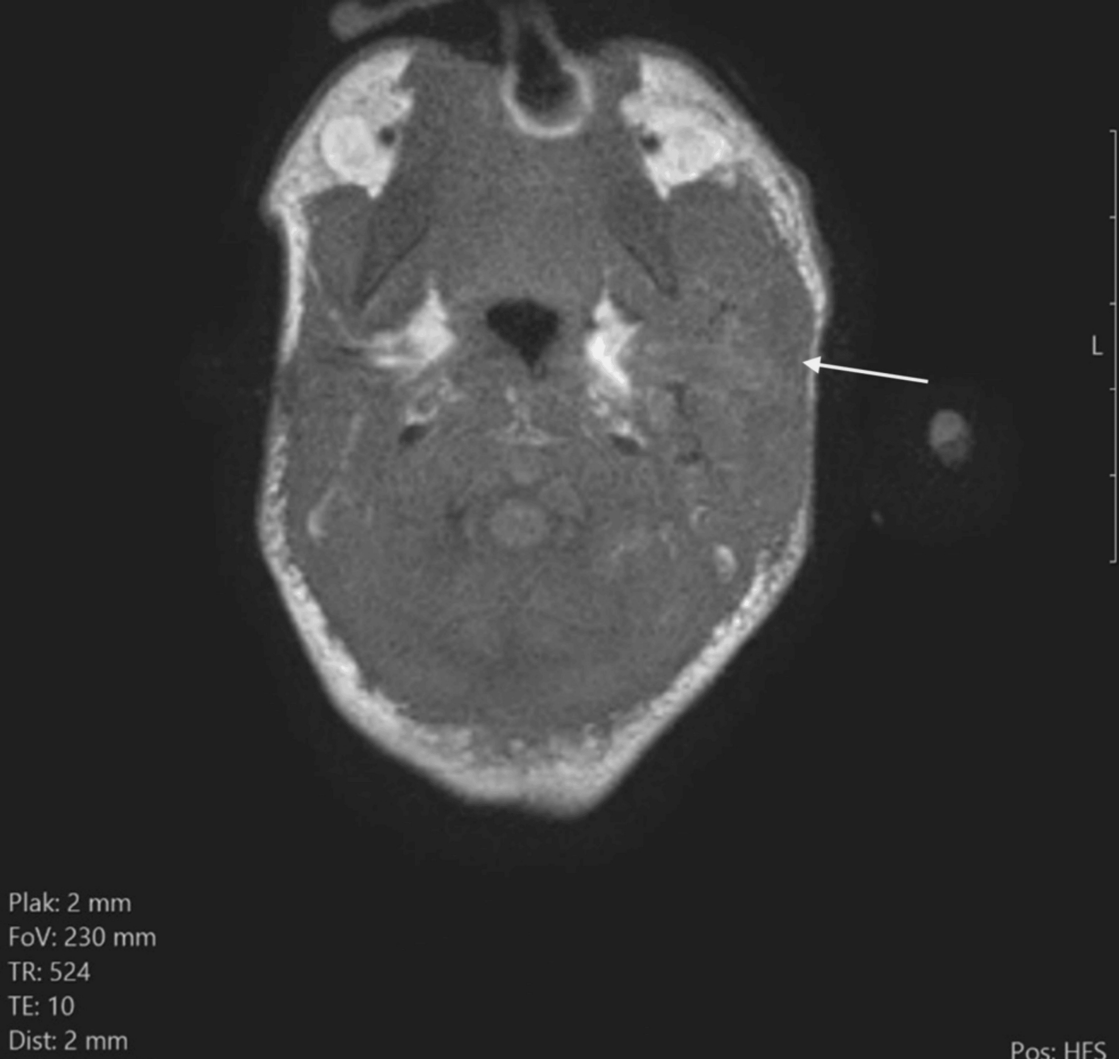
T1 TSE shows an iso-intense enlargement of the left parotid (white arrow) compared to the normal right side.

On T1 TSE images, the mass was iso-intense to the normal parotid on the right side (Figure 5).

**Figure 6:**
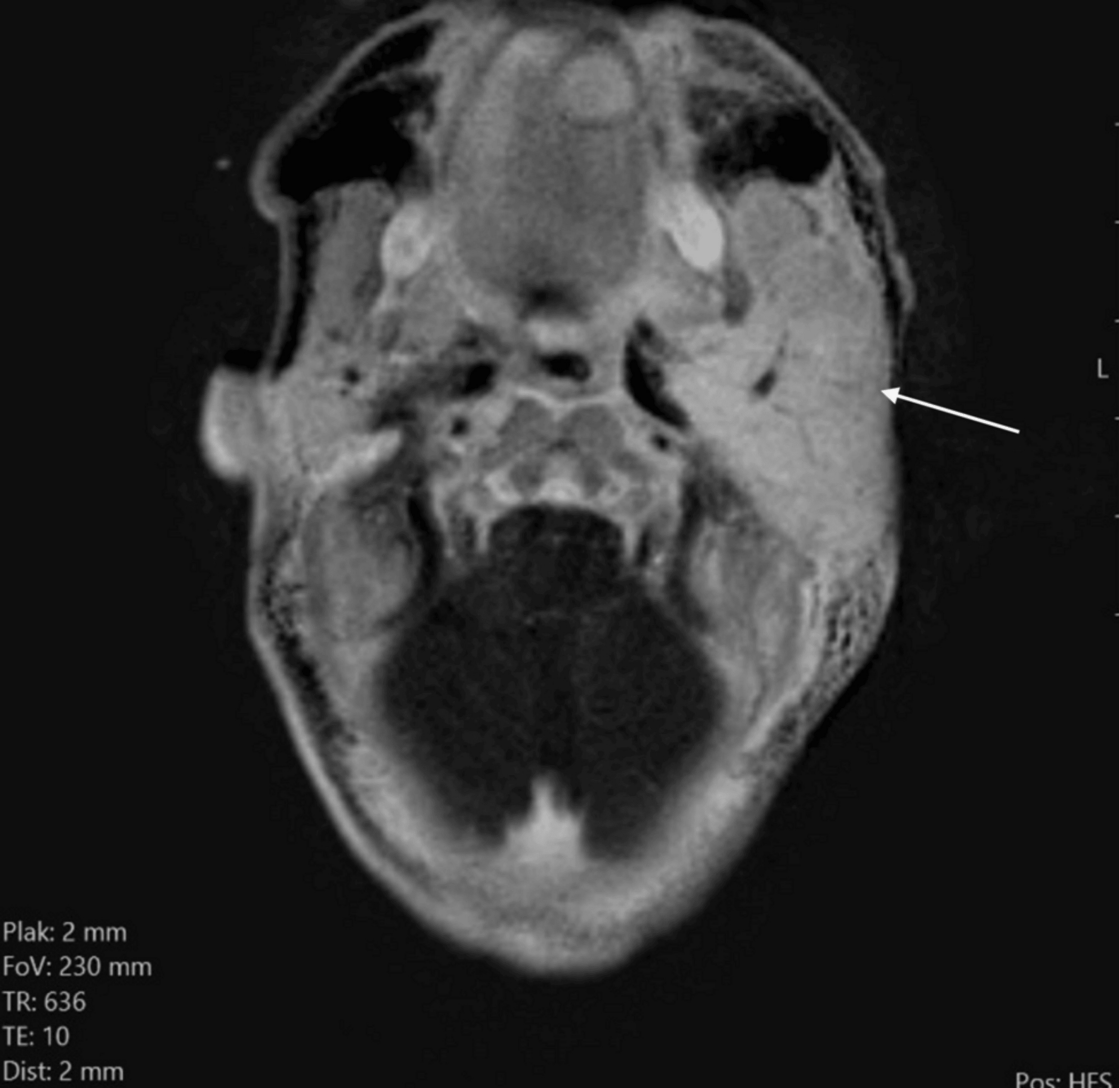
Axial substraction T1 TSE shows homogenous intense enhancement of the enlarged left parotid (white arrow).

On post-contrast subtraction T1-weighted images, the mass showed homogenous enhancement (Figure 6).

**Figure 7:**
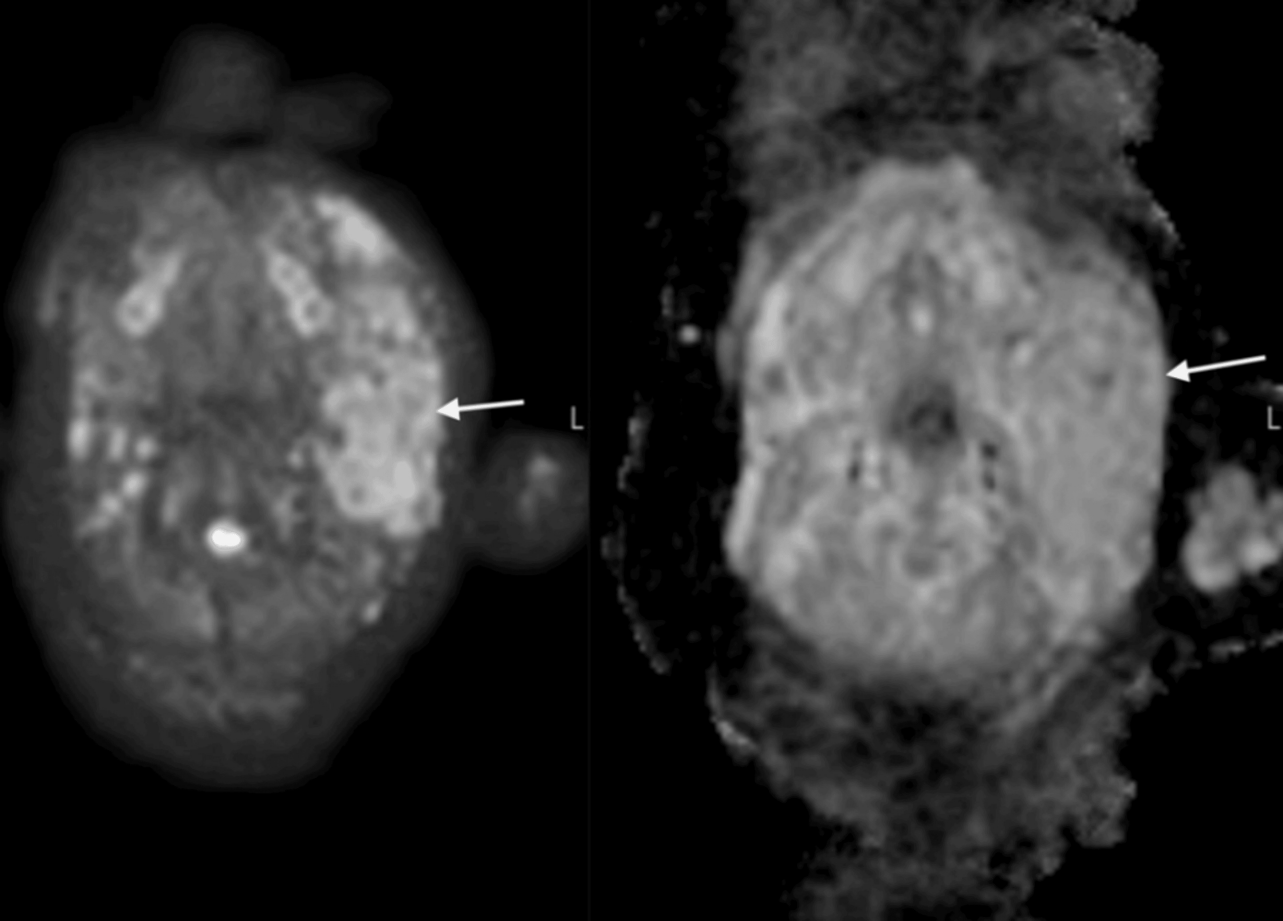
DWI shows T2 shine-through phenomenon of the left enlarged parotid (white arrows).

DWI demonstrated the T2 shine-through phenomenon of the enlarged parotid gland; there was no intralesional restricted diffusion (Figure 7).

These findings led to the final diagnosis of an infantile parotid hemangioma.

**Figure 8:**
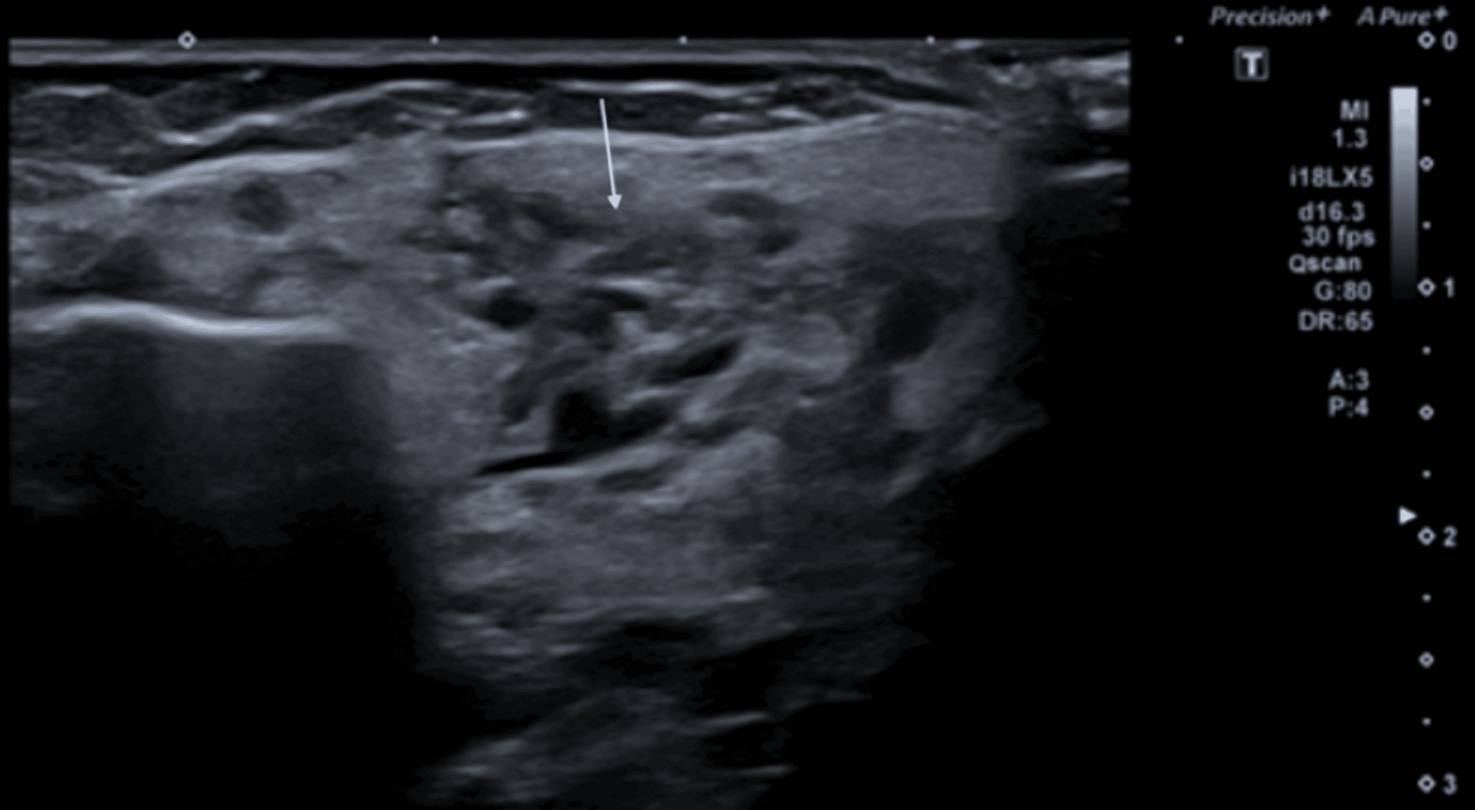
Follow-up ultrasounds demonstrated a good response, with complete regression of the parotid mass. The presence of a residual amorphous hypoechogenic area (white arrow) in the parotid gland is indicative of a residual lesion.

Further clinical evaluation identified two smaller cutaneous “strawberry” hemangiomas, on the back and on the left forearm. A subsequent ultrasound of the abdomen was negative for hepatic hemangiomas. Treatment with propranolol was initiated and increased according to a build-up schedule to a maximum dosage of 3 mg/kg/day. The treatment was well-tolerated, with no adverse events recorded. Tensions, glycemia, and potassium levels remained stable throughout the treatment period. Follow-up ultrasounds in the subsequent seven months demonstrated a good response, with complete regression of the parotid mass (Figure 8). The presence of a residual amorphous hypoechogenic area in the parotid gland is indicative of a residual lesion. The cutaneous hemangiomas slightly decreased in size, but remained visible during this period. 

The treatment was discontinued abruptly, approximately 10 months after the initial diagnosis. Subsequent follow-up, both clinical and ultrasound-based, is anticipated. If rebound growth occurs, the treatment will be reinitiated.

## Discussion

IPH are the most common parotid tumor in infancy, though overall it remains a rare entity among pediatric neck masses. IPH are typically benign and self-limiting. Accurate diagnosis is essential, as clinical presentation can mimic other conditions such as lymphadenitis (colli) or parotitis. This may lead to diagnostic uncertainty and, if misidentified, to delays in appropriate treatment and unnecessary interventions, such as biopsy or surgical excision.

Imaging is key to confirm the diagnosis of IPH, with ultrasound often serving as the first-line modality due to its availability and safety in neonates. Ultrasound typically reveals an iso- or hypoechoic, well-circumscribed, lobulated mass with fine linear septations and increased vascularity on Doppler [1,3]. The mass can replace or expand the parotid tissue [1]. However, in cases where ultrasound results are inconclusive or when further anatomical detail is needed, MRI can be used to further differentiate the lesion. On MRI, IPH look like lobulated homogenous T1-isointense masses with visible flow voids on T2-weighted images [1-3]. They are uniformly hyperintense on T2 FS and show intense, homogeneous enhancement post-contrast administration [1-3]. These hallmark features help distinguish IPH from other parotid or neck pathologies, especially in patients without associated skin lesions, as was evident in our case. Correlating imaging findings with clinical context is vital. In our patient, the lack of systemic signs of infection, normal inflammatory markers, and typical imaging features helped steer the diagnosis away from lymphadenitis toward IPH.

Although IPH are benign in nature, complications are not uncommon [6]. Ulceration is the most frequently reported issue, typically occurring during the early proliferative phase. Extension into adjacent structures is also common, while more severe complications such as airway obstruction and congestive heart failure are less frequently observed.

Management strategies for IPH have evolved dramatically since the introduction of propranolol therapy. Propranolol is now widely regarded as the first-line treatment due to its high efficacy and favourable safety profile [1,4,5,7,8]. Alternative treatments described in the literature include corticosteroids, imiquimod, vincristine, bleomycin A5, interferon-α, ACE inhibitors (captopril), laser therapy, and surgical excision [4]. The working mechanism of propranolol involves vasoconstriction, downregulation of angiogenic factors, and induction of apoptosis in endothelial cells [4,7,9]. Our patient demonstrated excellent clinical and radiological response to a conventional dosage of propranolol (2-3 mg/kg/day) over a mean duration time of 10 months, consistent with outcomes reported in the literature [4,5,7,8]. However, it has been reported that parotid hemangiomas may exhibit a slower response to propranolol and require a longer treatment duration compared to infantile hemangiomas at other anatomical sites [8]. Given the potential for spontaneous regression, conservative management is often sufficient, but in symptomatic or cosmetically concerning cases, treatment with propranolol has shown significant efficacy [1,4,5,7,8]. Clinicians should also be aware of the potential for rebound growth after discontinuation of therapy, as noted in several studies [4,5,7,8].

## Conclusions

IPH are the most common infantile parotid tumor. This case highlights the diagnostic value of ultrasound and MRI in identifying IPH. Recognizing the characteristic imaging features on ultrasound and MRI is key for an accurate diagnosis and differentiation from other pathologies, avoiding unnecessary interventions such as biopsy or surgical excision. It also reinforces the role of propranolol as a first-line therapy.
